# Percutaneous pedicle screw fixation was effective for bone regeneration after a huge vertebral defect due to intractable pyogenic spondylitis caused by methicillin-resistant *Staphylococcus aureus*: a case report

**DOI:** 10.1186/s13256-023-03942-w

**Published:** 2023-05-13

**Authors:** Masaki Tatsumura, Fumihiko Eto, Mikiro Kato, Katsuya Nagashima, Yosuke Takeuchi, Toru Funayama, Masashi Yamazaki

**Affiliations:** 1 Department of Orthopaedic Surgery and Sports Medicine, Tsukuba University Hospital Mito Clinical Education and Training Center/Mito Kyodo General Hospital, 3-2-7 Miyamachi, Mito, Ibaraki 310-0015 Japan; 2 Department of Orthopaedic Surgery, Faculty of Medicine, University of Tsukuba, Tsukuba, Japan; 3 Department of Infectious Diseases, Faculty of Medicine, University of Tsukuba, Tsukuba, Japan

**Keywords:** Methicillin-resistant *Staphylococcus aureus* (MRSA), Huge vertebral defect, Percutaneous pedicle screw (PPS), Bone regeneration, Infection control, Non-bone transplantation, Case report

## Abstract

**Background:**

Pyogenic spondylitis by methicillin-resistant *Staphylococcus aureus* (MRSA) is known to be intractable. In the past, the insertion of an implant into infected vertebra was considered contraindicated in affected patients because it may exacerbate the infection, but there are increasing numbers of reports indicating the usefulness of posterior fixation to correct instability and alleviate infection. Bone grafting is often required to repair large bone defect due to infection, but free grafts can exacerbate infection and are controversial.

**Case presentation:**

We present the case of a 58-year-old Asian man with intractable pyogenic spondylitis who had repeated septic shocks due to MRSA. Back pain from repeated pyogenic spondylitis caused by a huge bone defect in L1–2 rendered him unable to sit. Posterior fixation by percutaneous pedicle screws (PPSs) without bone transplantation improved spinal stability and regenerated bone in the huge vertebral defect. He regained his activities of daily living, had no reoccurrence of pyogenic spondylitis nor bacteremia, and was completely cured of the infection without antibiotics after removal of all screws.

**Conclusions:**

For intractable MRSA pyogenic spondylitis with instability accompanied by a huge bone defect, posterior fixation using PPSs and administration of antibacterial agents stopped the infection, allowed the bone to regenerate, and recovered the patient’s activities of daily living.

## Background

The incidence of pyogenic spondylitis has been increasing as a result of population aging and compromised hosts. Early diagnosis and identification of the causative organism, and administration of appropriate antibacterial agents are important for the treatment of pyogenic spondylitis. Conservative treatment with antibiotics is recommended for 6 weeks, and surgical treatment is not required if neurological compromise, extensive bone destruction, epidural abscess formation, failure of nonsurgical treatment, or intractable back pain are avoided [1].

Frequent causative organisms of pyogenic spondylitis are *Staphylococcus aureus* (48%), *Escherichia coli* (11%), streptococci (9.4%), *Coagulase-negative* staphylococci (2.7%), and *Pseudomonas aeruginosa* (2.0%) [2]. Among them, pyogenic spondylitis as a result of methicillin-resistant *Staphylococcus aureus* (MRSA) is known for its intractability.

Instability of the spinal column is also a cause of intractable pyogenic spondylitis. Percutaneous pedicle screws (PPSs) can be used to stabilize the spinal column [3]. A small incision can be used in minimally invasive surgery for posterior fixation and decompression of peripheral muscles compared with a more invasive conventional posterior approach. Because it is less invasive than the conventional method, PPSs are used widely for elderly people with poor general condition.

In this report, we present a case of pyogenic spondylitis by MRSA that was resistant to conservative treatment with antibiotics and resulted in a huge bone defect in the vertebral bodies due to repeated pyogenic spondylitis by the MRSA infection. We performed posterior fixation by PPS fixation to stabilize the patient’s vertebrae over the huge bone defect and ultimately obtained complete freedom from infection.

## Case report

**Fig. 1:**
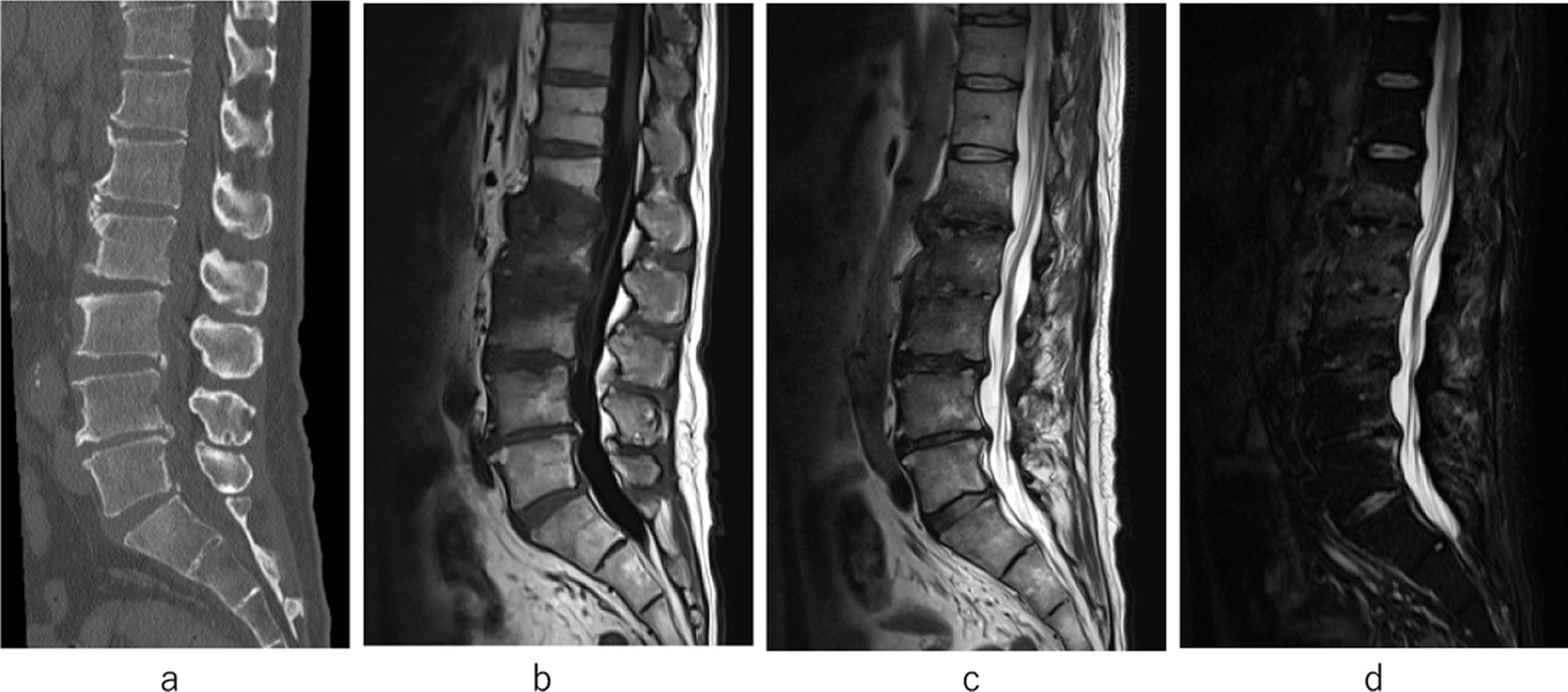
At the first admission. Endplate appeared normal on computed tomography (**a**). Low signal changes in the L1-2 and L2-3 disc spaces are shown on T1-weighted magnetic resonance images (**b**) and T2-weighted MR images (**c**). There was no change on short tau image recovery in MR imaging (**d**)

A 58-year-old Asian man with a 20-year history of schizophrenia presented with low back pain that appeared without any apparent trauma. There was no history of immunosuppressive diseases, such as diabetes or malignant disease, and the patient had never been treated with corticosteroids. On the 3^rd^ day after the onset of low back pain, he was found lying down at his home and was transported to the emergency clinic of another hospital. Although conservative treatment was started with a diagnosis of MRSA septic shock, the patient was transferred to our hospital 14 days after the onset because of poor infection control and persistent shock (1st admission). The results of blood examinations showed 9700 white blood cells (WBC) per μL, and C-reactive protein (CRP) 9.38 mg/dL. Three blood cultures were collected, and all three sets were positive for MRSA. The patient could not even sit up because of the intense pain. We diagnosed the patient with pyogenic spondylitis of the L1, L2, and L3 vertebrae, and arthritis of his left knee with bacteremia. At first, there was an apparently normal endplate seen on CT and a signal change in the L1–2 and L2–3 disc spaces on MRI (Fig. 1a–d). We selected vancomycin (VCM) for 6 weeks and sequentially daptomycin (DAP) for 8 weeks as intravenous antibacterial agents. After the inflammatory findings on the blood examination had decreased (WBC 6000 cells/μL and CRP 4.04 mg/dL) and the image showed no abscess in any lesion including the lumbar vertebrae and knee, he was able to walk with a cane and was discharged from our hospital.

**Fig. 2:**
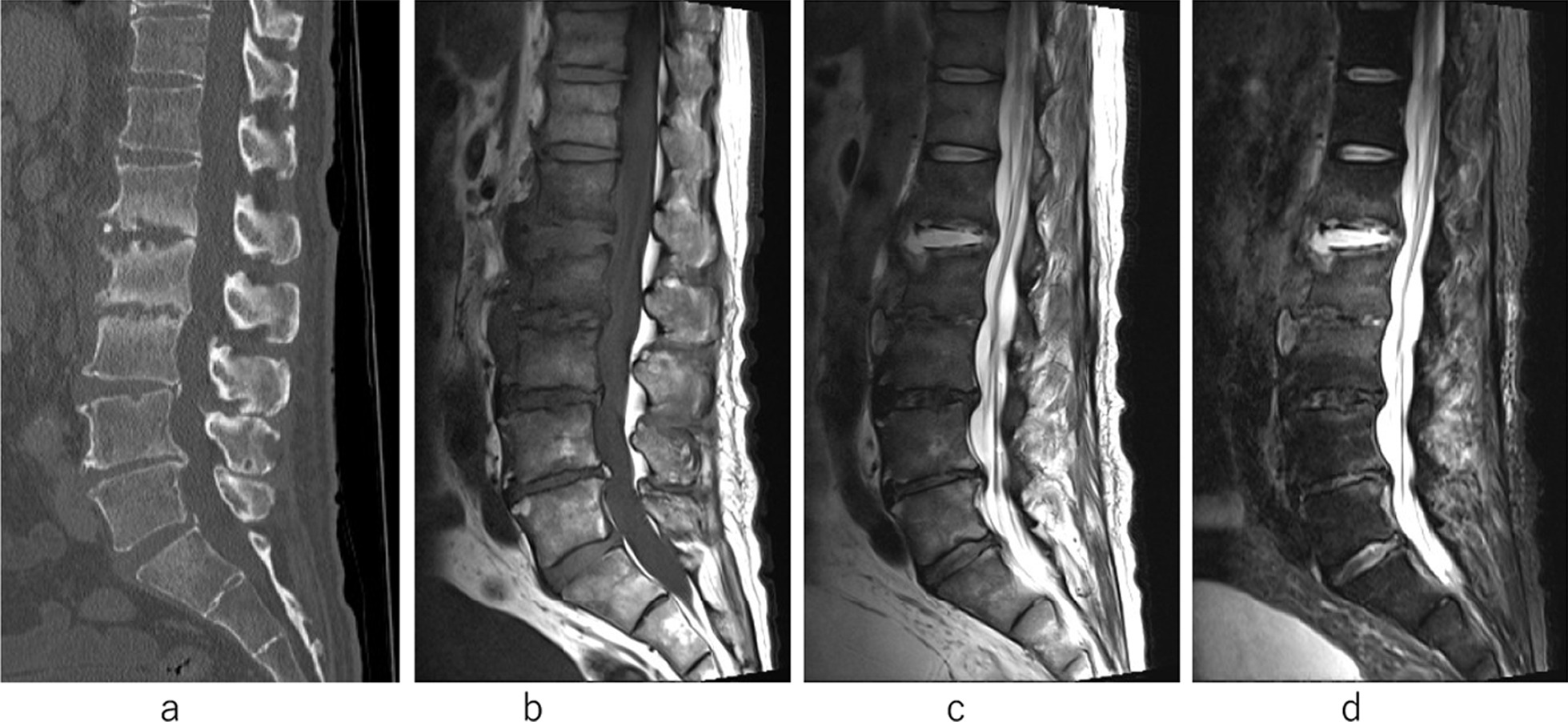
At the second admission. There was slight endplate irregularity in the L1–2 and L2–3 disc spaces on computed tomography (**a**). There is fluid signal in the L1–2 intervertebral space, which was low intensity on T1-weighted magnetic resonance images and high intensity on T2-weighted MR images and short tau image recovery in MRI (**b**–**d**)

However, low back pain appeared again 6 months after the initial onset, and CT showed slight changes of endplates of L1–2 and L2–3 and MRI showed fluid retention in the L1–2 intervertebral disc space (Fig. 2a–d). His WBC count was 15100 cells/μL and CRP was 24.87 mg/dL. Two blood cultures were collected and both sets were positive for MRSA. He had moderate back pain but could walk with a cane. On the diagnosis of reoccurrence of pyogenic spondylitis at L1 and L2 with bacteremia, intravenous VCM for 4 weeks and sequentially DAP for 4 weeks was started (2nd admission). Oral administration of minocycline (MINO) was continued when the signs of infection subsided (WBC count 5100 cells/μL and CRP 2.26 mg/dL). He was discharged from the hospital, and could walk without back pain.

**Fig. 3:**
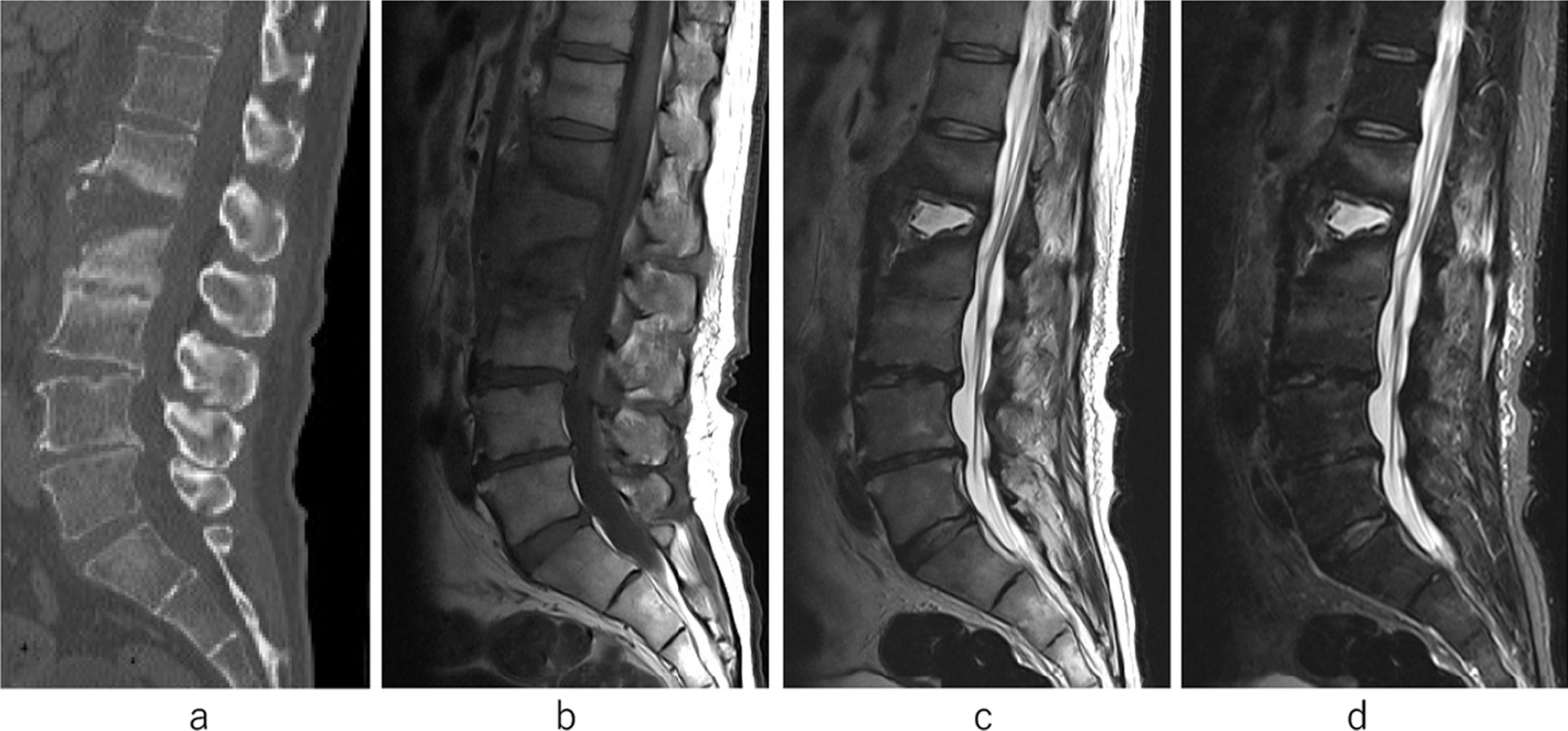
At the third admission. There was bone absorption in L1–2 and bone union in L2–3 vertebrae on computed tomography (**a**). The fluid signal in the L1–2 disc space was expanded on T2-weighted MR images and short tau image recovery of MRI. Low signal intensity in the L1–2 and L2-3 disc spaces on T1-weighted magnetic resonance images remained (**b**–**d**)

However, 9 months after the onset, low back pain recurred and he represented at our hospital emergency clinic (3rd admission). Progress of vertebral body destruction of L1 and L2 on CT and bone marrow edema around the L1–2 endplate on MRI were observed. His WBC count was 5600 cells/μL and CRP concentration was 12.38 mg/dL. Blood cultures were negative, but he was unable to walk due to severe back pain. Oral MINO was discontinued, and we diagnosed the patient with third pyogenic spondylitis of L1and L2 without bacteremia (Fig. 3a–d). After intravenous administration of DAP for 8 weeks, inflammation abated (WBC count 5000 cells/μL and CRP 0.63 mg/dL). We changed to oral administration of MINO and continued this treatment.

**Fig. 4:**
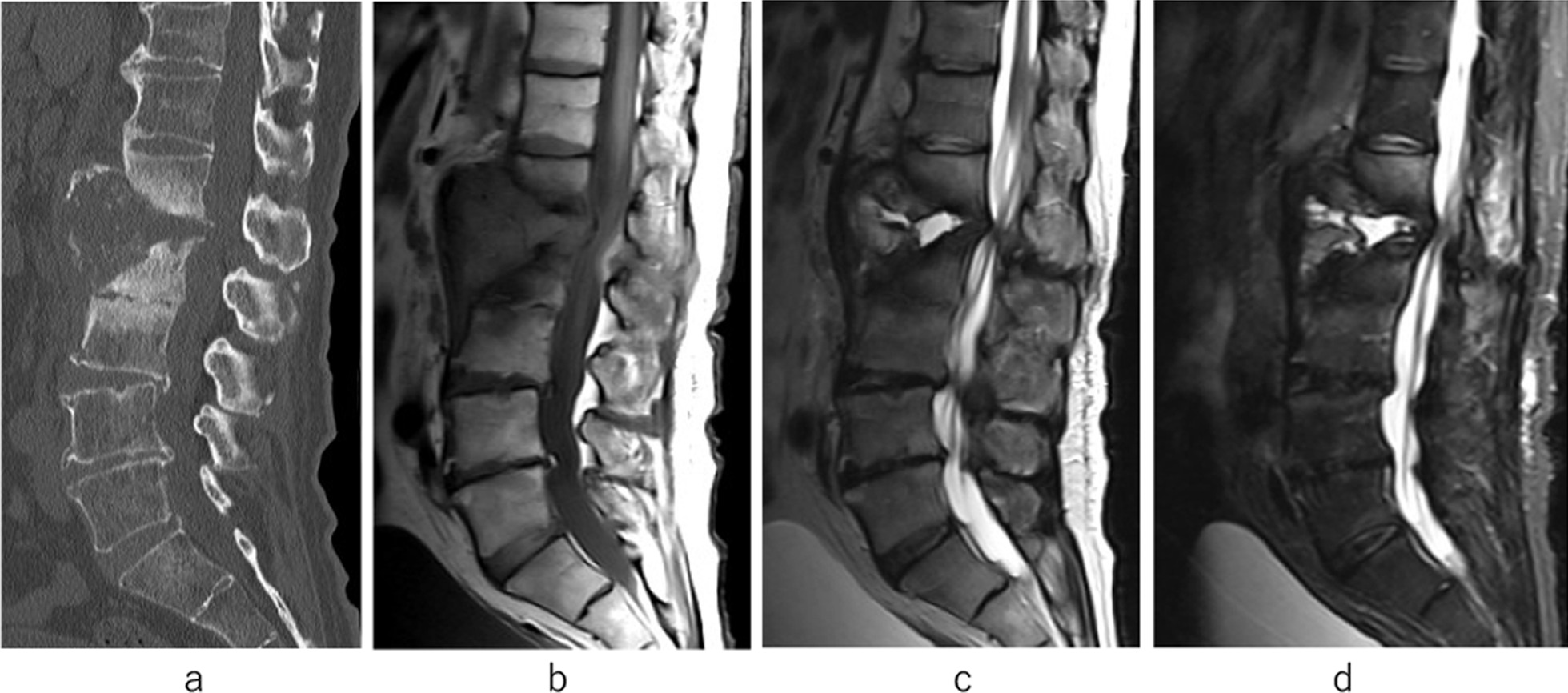
At the 4th hospitalization. A huge bone defect in L1 and L2 is shown on computed tomography (**a**). MRI shows an abscess in the L1–2 disc space that was enlarged toward the anterior (**b**–**d**)

**Fig. 5:**
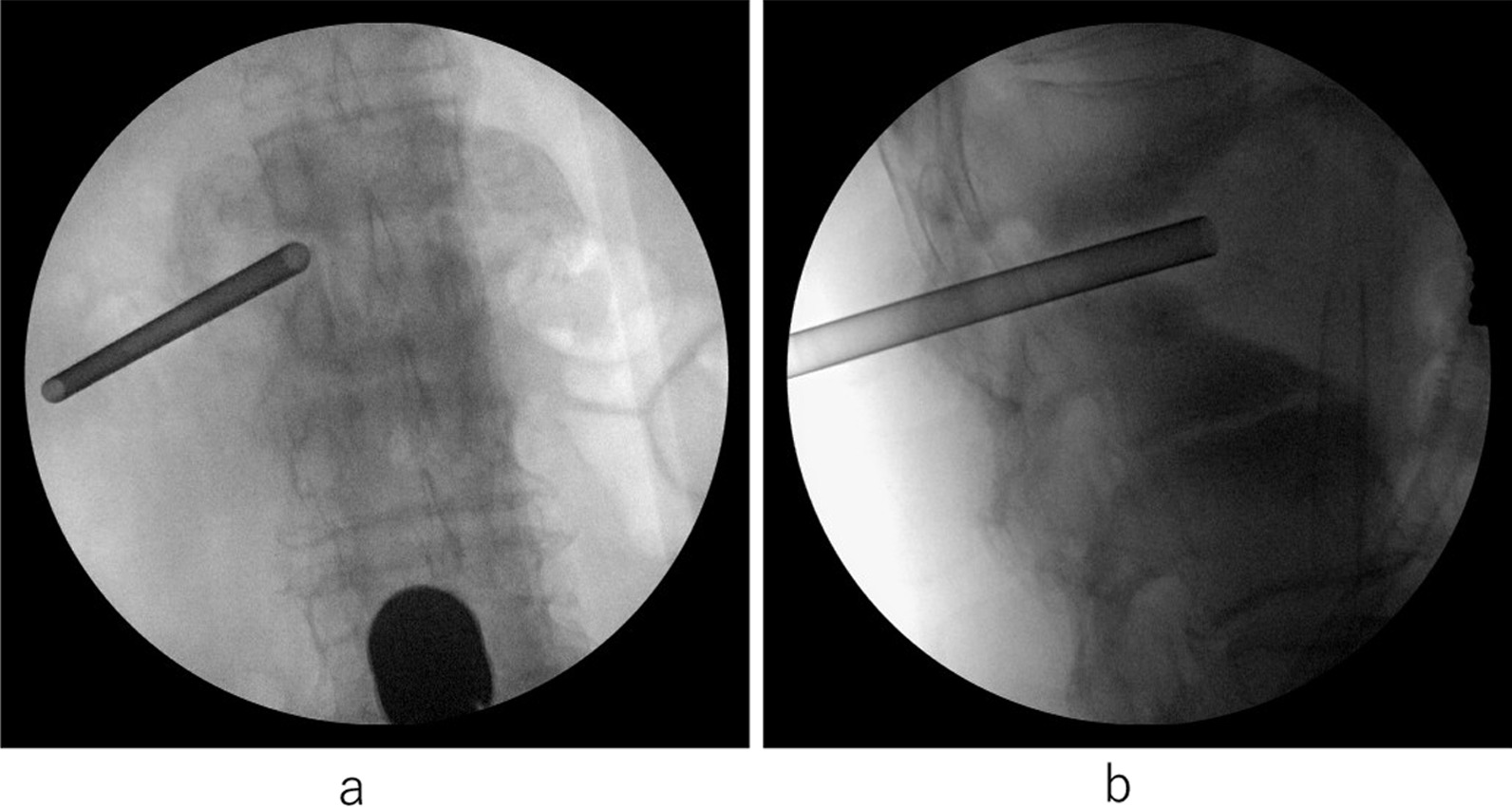
Full endoscopic irrigation of the L1–2 disc space (**a**, **b**)

**Fig. 6:**
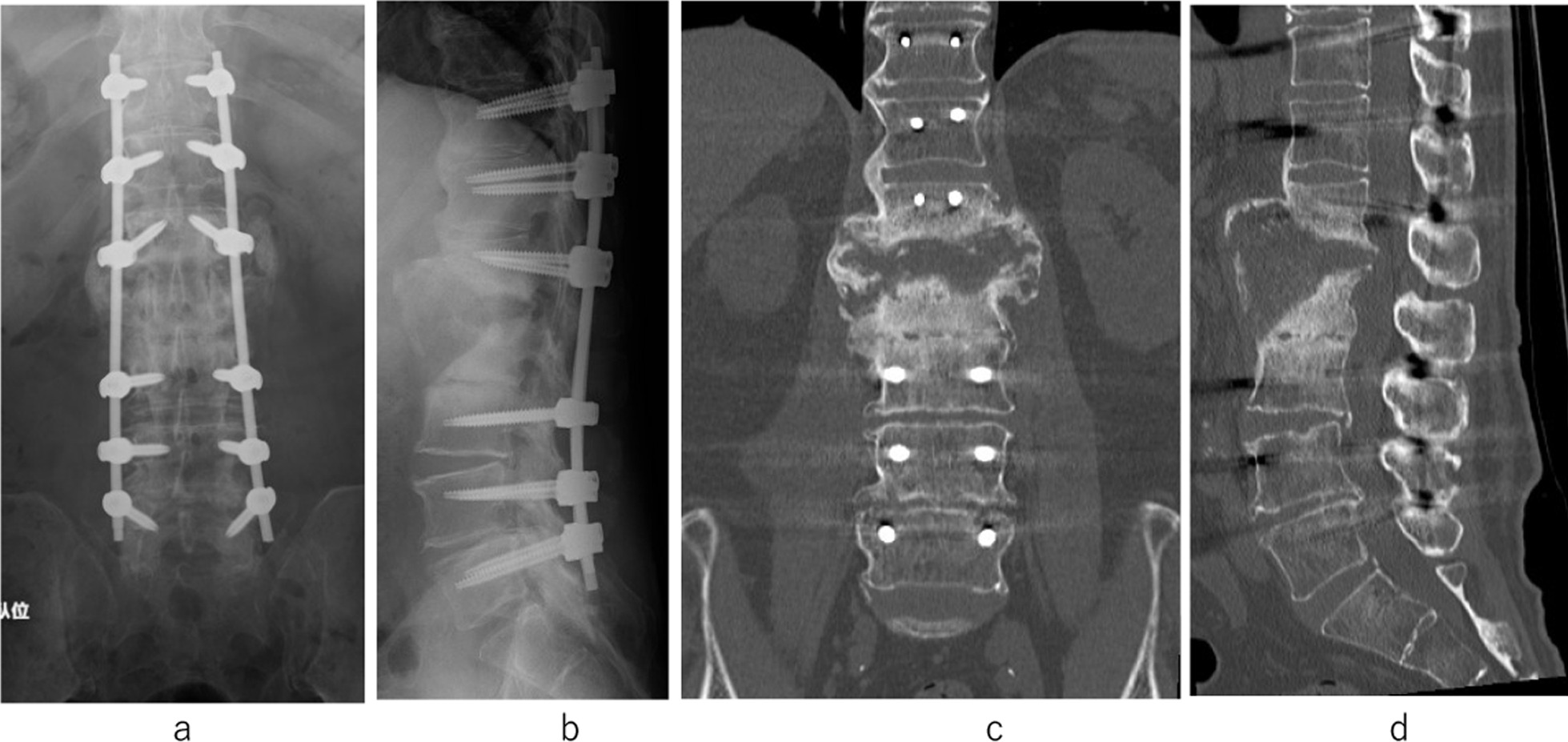
Plain X-ray imaging and computed tomography showing posterior fixation with percutaneous pedicle screws inserted in T11, T12, L1, L3, L4, and L5 vertebrae (**a**–**d**)

Although the patient was once again in remission, low back pain reappeared 15 months after the initial onset. He was unable to walk due to severe back pain. Imaging examination revealed a huge bone defect in the L1–2 disc space with a vertebrae defect. A blood examination showed a white blood cell count of 5500/μL, which was within the normal range, but a slight increase in CRP concentration to 0.33 mg/dL was observed. Blood cultures were negative. No other findings indicating organ damage were found. To treat the repeated pyogenic spondylitis of L1 and L2, DAP was administered after the fourth hospitalization (4th admission). However, the bone defect between the vertebral bodies of L1 and L2 had become large, which caused spinal instability between the L1 and L2 vertebrae with a huge abscess (Fig. 4a–d). We performed percutaneous endoscopic irrigation (Fig. 5a, b), but the inflammation continued. We considered that one of the causes of the infection reoccurrence in this case was the instability resulting from the huge vertebral bone defect. As a treatment strategy, we chose to perform posterior fixation using PPSs, which were inserted into T11, T12, L1, L3, L4, and L5 vertebrae 16 months after the initial onset of the low back pain (Fig. 6a–d). Because they were almost entirely defective, we abandoned inserting screws into L2. Autologous bone grafting was not performed, nor did we reconstruct the anterior strut.

**Fig. 7:**
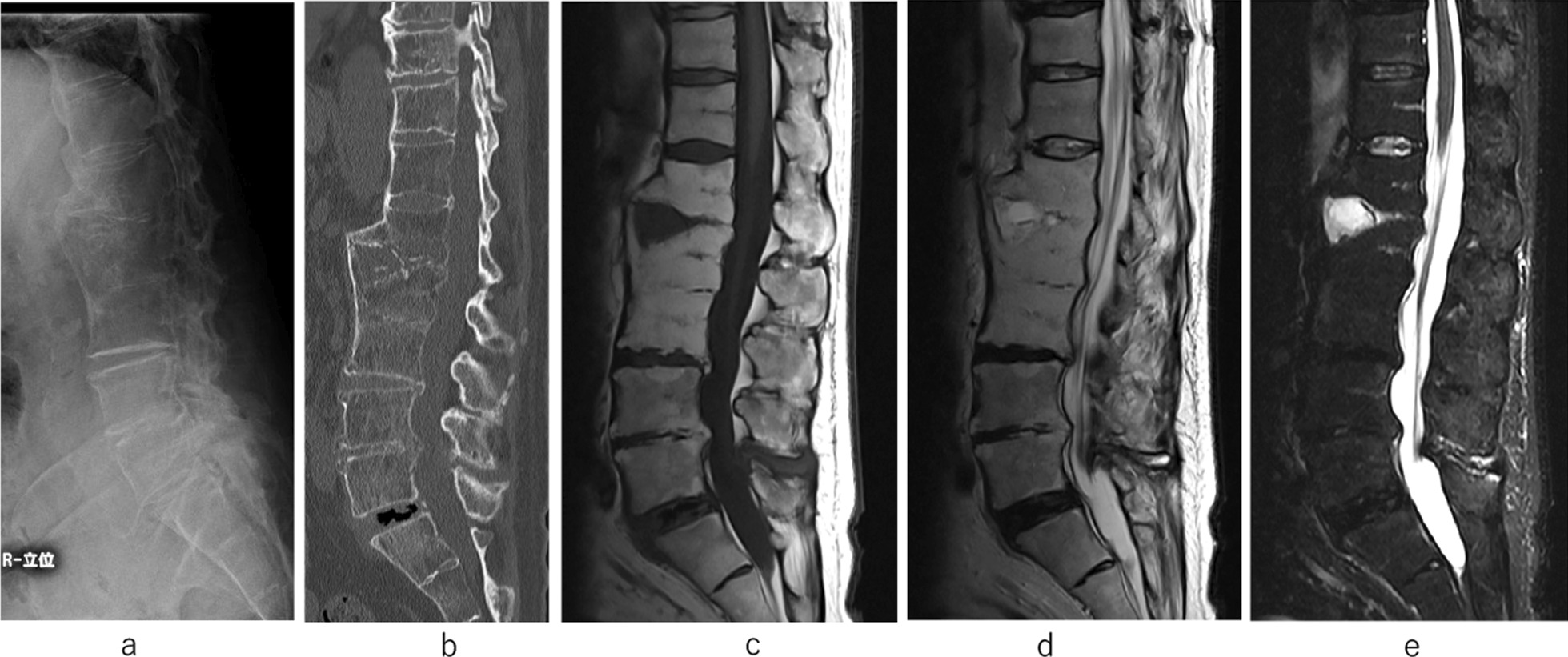
Plain X-ray imaging and computed tomography after screw removal. computed tomography showing bone regeneration at L1 and L2 at 1 year after surgery (**a**, **b**). MRI shows no bone marrow edema or bone cyst in the L1–2 disc space (**c**–**e**)

**Fig. 8:**
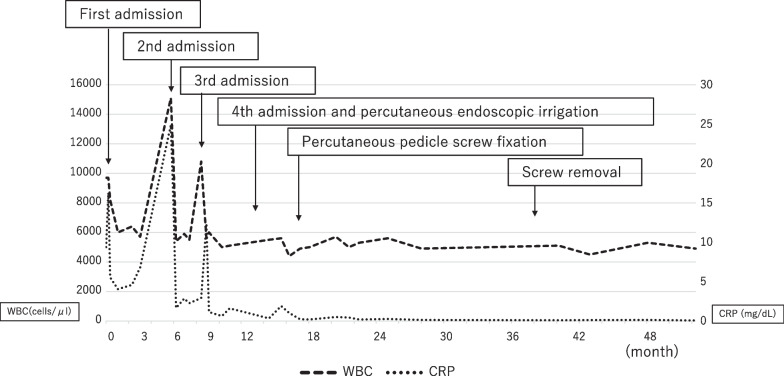
Time course of white blood cell counts and C-reactive protein concentrations. The timing of each admission and the timing of events for percutaneous endoscopic irrigation, percutaneous pedicle screw fixation, and screw removal are noted

After the operation, the patient wore a soft brace and started to walk as physiotherapy. After intravenous administration of DAP for 8 weeks, oral administration of MINO was continued for 2 years. Two years after the surgery, bone regeneration was observed between the L1 and L2 vertebral bodies where bone defect had occurred, there was no progression of the abscess, and the patient could walk independently without low back pain. Therefore, we decided to remove the instrumentation, which may potentially cause delayed infection. After removal of all screws, the administration of MINO was postponed. There was no reoccurrence of pyogenic spondylitis and his spinal structure was stabilized for 2 years after ceasing all antibiotic agents. Plain X-ray imaging and CT after screw removal showed bone regeneration at L1 and L2 at 1 year after surgery (Fig. 7a, b). MRI showed no bone marrow edema or bone cyst in the L1-2 disc space (Fig. 7c–e). The changes in WBC count and CRP concentration with time are shown (Fig. 8).

## Discussion

According to the guidelines of the American Society of Infectious Diseases [4], an appropriate antibacterial drug is recommended for 6 weeks as a treatment of pyogenic spondylitis. The guidelines clearly state that the spine should be evaluated by MRI. Surgical treatment, including fusion is recommended if the patient has neurological symptoms or instability.

Most cases of pyogenic spondylitis diagnosed early result in a good clinical course by conservative treatment [5]. Bed rest should be used effectively to prevent vertebral destruction due to weight bearing on the spinal lesion because it is important to maintain the biomechanical stability of the spinal column. Multilevel disease of 3 or more vertebrae, resistant bacterial infections such as MRSA, appearance of neurological symptoms due to an epidural abscess, and low Hb level are a result of conservative treatment resistance and predictors of a poor prognosis of pyogenic spondylitis [6]. One indication for surgery is serious vertebral destruction with instability [4]. Reoccurrence despite appropriate antimicrobial therapy is another [7].

The patient with this case of MRSA infection showed multiple reoccurrences despite conservative treatment with antibacterial agents for 6 weeks or longer at each admission to hospital. The instability of his spine progressed after repeated infection and surgical treatment was indicated. After his spinal structure was stabilized, infection was not present and he was free from any antibiotic agent for one year. PPS fixation has the effect of not only of stabilizing the spine, but was also useful to control the general infection.

A risk factor for pyogenic spondylitis caused by multidrug-resistant bacteria, such as MRSA, is immunosuppression [8]. Al-Nammari et al. reported that 38% of patients with MRSA spondylitis died within 6 months of diagnosis and 8% needed continuous treatment [9]. In the present case, we could not withdraw the antibiotic because of the risk to preservation of the implant and reoccurrence of the infection. We considered that the infection would not recur if the implant was removed and the antibiotic was eventually terminated.

The huge defect of the anterior and middle column presented because of bone destruction by repeated pyogenic spondylitis. As the bone defect was large, we also considered performing anterior fixation. Aortic rupture has been reported when the aorta and the reservoir were in close proximity [10], and it was considered that the aorta has a fragile vessel wall. We chose posterior fixation alone without anterior strut reconstruction, resulting in sufficient bone regeneration. Bone grafting was not performed because free grafted bone may be a source of infection. Scaffolding remains even in bone decalcified by infection, and we considered the vertebra would be remineralized if the infection was controlled.

We used PPSs for this case because one of their advantages is that back muscle damage is minimized. Because of repeated hospitalization his back muscle was atrophied, we were obliged to perform stabilization that was minimally invasive.

We continued the oral antibiotics for several months to avoid surgical site infection. Because bone regenerated rapidly in this case, we decided to remove the implant. After removal, the oral antibiotics were stopped and no reoccurrence has presented for at least 1 year.

## Conclusion

For intractable MRSA pyogenic spondylitis accompanied by a huge bone defect, posterior fixation using PPSs and administration of antibacterial agents stopped the infection, allowed the bone to regenerate, and recovered his activities of daily living. After removal of all screws, there was no reoccurrence of pyogenic spondylitis and his spinal structure was stabilized without all antibiotic agents.

## Data Availability

The data analyzed in the current case are available from the corresponding author on reasonable request after anonymization so that the patient cannot be identified.

## Abbreviations

MRSA: Methicillin-resistant *Staphylococcus aureus*
PPS: Percutaneous pedicle screw
VCM: Vancomycin
DAP: Daptomycin
MINO: Minocycline
